# The importance of plasmonic heating for the plasmon-driven photodimerization of 4-nitrothiophenol

**DOI:** 10.1038/s41598-019-38627-2

**Published:** 2019-02-28

**Authors:** Radwan M. Sarhan, Wouter Koopman, Roman Schuetz, Thomas Schmid, Ferenc Liebig, Joachim Koetz, Matias Bargheer

**Affiliations:** 1 University of Potsdam, Institute of Physics and Astronomy, Karl-Liebknecht-Str. 24-25, 14476 Potsdam, Germany; 2 University of Potsdam, Institute of Chemistry, Karl- Liebknecht- Straße 24-25, 14476 Potsdam, Germany; 3 School of Analytical Sciences Adlershof (SALSA), Humboldt-Universität zu Berlin, Albert-Einstein-Str. 5–9, 10099 Berlin, Germany; 4 Max Planck Institute of Colloids and Interfaces, Department of Biomaterials, Am Mühlenberg 1, 14476 Potsdam, Germany; 5 Federal Institute for Materials Research and Testing, Richard-Willstätter-Str. 11, 12489 Berlin, Germany; 6 Helmholtz Zentrum Berlin, Albert-Einstein-Str. 15, 12489 Berlin, Germany

## Abstract

Metal nanoparticles form potent nanoreactors, driven by the optical generation of energetic electrons and nanoscale heat. The relative influence of these two factors on nanoscale chemistry is strongly debated. This article discusses the temperature dependence of the dimerization of 4-nitrothiophenol (4-NTP) into 4,4′-dimercaptoazobenzene (DMAB) adsorbed on gold nanoflowers by Surface-Enhanced Raman Scattering (SERS). Raman thermometry shows a significant optical heating of the particles. The ratio of the Stokes and the anti-Stokes Raman signal moreover demonstrates that the molecular temperature during the reaction rises beyond the average crystal lattice temperature of the plasmonic particles. The product bands have an even higher temperature than reactant bands, which suggests that the reaction proceeds preferentially at thermal hot spots. In addition, kinetic measurements of the reaction during external heating of the reaction environment yield a considerable rise of the reaction rate with temperature. Despite this significant heating effects, a comparison of SERS spectra recorded after heating the sample by an external heater to spectra recorded after prolonged illumination shows that the reaction is strictly photo-driven. While in both cases the temperature increase is comparable, the dimerization occurs only in the presence of light. Intensity dependent measurements at fixed temperatures confirm this finding.

## Introduction

The optical excitation of metal nanoparticles allows novel chemical transformation pathways, unknown in conventional chemical synthesis^1–3^. The mechanism driving these reactions is however still a source of a much controversy. In particular, the relative influences of plasmonic nanoscale heating^4–6^ compared to the generation of energetic electrons has been debated^7–10^.

Plasmons are coherent oscillations of the nanoparticle conduction-band electrons. Energetic electrons are formed as a consequence of the dephasing of this coherent electron motion – known as plasmon relaxation^11^. Electron-electron scattering subsequently leads to a thermalization of the electron gas, described by the Fermi-Dirac distribution with elevated temperatures^6^. Some of these “hot” energetic electrons might have sufficient energy to populate the orbitals of molecules residing on the particle surface and initiate chemical transformations. This mechanism has been known in the femto-chemistry community for a long time as desorption induced by electron transfer (DIET). It is often considered as primary source for plasmon driven chemistry as well. In femtosecond time-resolved experiments, cooling of the electrons by electron-phonon coupling is observed after few picoseconds, where the energy exchange between the electrons and the phonon mode of the nanoparticles results in an increase of the nanoparticle lattice temperature^12–14^. This heat is dissipated to the surrounding environment on a longer timescale^11^. An important question is whether this excess heat plays a significant role for plasmon driven reactions.

**Figure 1:**
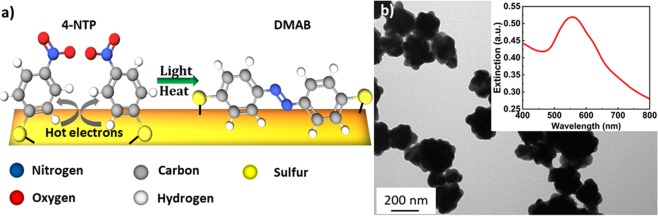
(**a**) Schematic of the chemical structures and the reaction. (**b**) TEM micrograph of gold nanoflowers (GNFs). The inset in (**b**) shows the extinction spectrum of the GNFs.

The dimerization of 4-nitrothiophenol (4-NTP) or 4-aminothiophenol (4-ATP) into 4,4′-dimercaptoazobenzene (DMAB) is considered a model example of plasmon driven reactions (Fig. 1a)^15–18^. Osawa *et al*. observed already in 1994 that SERS spectra of 4-NTP and 4-ATP molecules adsorbed on metal nanoparticles differed remarkably from Raman spectra in bulk powder^19^. It took about 15 years to realize that the molecules undergo a dimerization reaction and that the spectral changes are not the evidence of the chemical-enhancement mechanism of SERS^20^. The presence of DMAB was in the following time experimentally confirmed by surface mass spectrometry^21^.

However, these reactions are still a point of debate, since the mechanism of the reaction, and in particular the relative influence of energetic electron generation and nanoscale heating, is unclear^22^. For instance, dimerization of 4-ATP was explained to be driven by the plasmonic-heating process with assistance of the oxygen as the oxidizing agent^23^. However, the reaction was completely inhibited when the ammonium oxalate was added to the reaction medium as a hole-capturer. This suggests that the reaction is driven by a charge-transfer mechanism injecting electrons form the particle to the reactant^24^. On the other hand, *Kwan Kim* and co-worker have measured SERS spectra of 4-NTP adsorbed on silver nanoparticle-substrate at liquid nitrogen temperature (77 K)^8^. The authors showed that hot electrons were efficiently generated at this temperature while they did not observe any reaction signatures, which raised the debate about the role of the operating temperature. It was claimed that either the hot electrons did not have enough energy to reach the molecular orbitals of the adsorbate or the adsorbed molecules might be trapped in the ice crystal which cannot form the dimer form^8^. We may agree with the first reason since the SERS spectra of the 4-ATP molecules showed the vibrational modes of DMAB under the same conditions.

Recently, *Golubev et al*. claimed that plasmonic heating is the key of the plasmonic reaction of 4-NTP^7^, while around the same time *Keller and Frontiera* presented evidence produced by ultrafast Raman thermometry that heating is not a primary mechanism for plasmon-driven photocatalysis of 4-NTP^25^. Their experiments suggest that the temperature of the reactant rises only moderately by few ten Kelvin and the heat is dissipated within a few ps. However, no evidence of a substantial formation of products was given so it remains unclear if the reaction conditions were reached in these experiments.

On the other hand, we recently reported an increase of the nanoparticle lattice temperature of about 100 K during irradiation with a CW laser, which was detected by x-ray diffraction monitoring of the thermal expansion^26^. Other publications discuss that a temperature of 150–220 °C leads to bubble-formation in the solvent^27^. Such high temperatures caused by the plasmonic heating were not only reported to initiate chemical reactions of the adsorbed molecule^4^, but also to enhance the rate of hot electron-driven reactions^10^. For instance, the visible light-driven degradation of the methylene blue dye was reported on Au-ZnO NRs system^28^. The authors found that under resonant excitation of the system, the local temperature reached to 300 °C, which significantly increased the quantum yield of the degradation. Static Raman thermometry of plasmon driven charge injection into methylene blue even suggest molecular vibrational temperatures of up to 1000 K^29^.

In this contribution to the debate, we monitor the dimerization reaction of 4-NTP adsorbed on gold nanoflower-modified silicon substrates at different operating temperature. We show that the rate of the reaction depends on the operating temperature and on the intensity, which on the one hand increases the temperature, but on the other hand increases the number of hot carriers that initiate the reaction. From the Anti-Stokes-Stokes-ratio we measure the *in-situ* molecular and nanoparticle temperature induced by light and find a significant light induced heating of both. Moreover, the average DMAB product molecules are clearly much hotter than the average nanoparticle temperature. We conclude that DMAB forms preferentially at hot spots of the plasmonic excitation SERS sensing. Gradually increasing the operating temperature up to the damage threshold of the sample structure while measuring SERS spectra with very low laser power and very short integration time (resembling the dark environment) we do not observe the dimerization reaction. This is consistent with a plasmon-driven dimerization reaction of the 4-NTP which is mainly initiated by a hot-electron-transfer process, while the high temperature induced by the light only enhances the rate of the reaction. This finding is corroborated by changing the intensity by two orders of magnitude.

## Material and Methods

### Materials

Hydrogen tetrachloroaurate (III) trihydrate (HAuCl_4_.3H_2_O), 4-nitrothiophenol (4-NTP), and N-(2-Hydroxyethyl)piperazine-N′-(2-ethanesulfonic acid) (HEPES) were purchased from Sigma-Aldrich and used without further purification. Aqueous solutions of the chemicals were prepared using deionized water generated by a Millipore-Q system.

### Synthesis of gold nanoflowers (AuNFs)

Irregularly shaped gold nanoparticles, previously termed AuNFs in literature, were prepared according to the established procedure^30,31^. Briefly, 200 μl of 100 mM of HEPES buffer solution (pH 7.4 + 0.5) were thoroughly mixed with 1.8 mL water for 5 min. 40 μl of 25 mM aqueous solution of HAuCl4 was then quickly added. The final mixture was left undisturbed for 2 h, during which the color turned from pale yellow to colorless and finally to dark blue confirming the reduction process. The AuNFs were washed several times by centrifugation and finally dispersed in 500 μl water.

### Fabrication of SERS-substrate

1 cm^2^ pieces of silicon wafers were cleaned by immersing them in a solution of 30 wt % H_2_O_2_ and 30 wt % H_2_SO_4_ for 1 hour. The wafers were then washed in ethanol and water several times before being used. 50 μl of the AuNFs solution was then deposited on the silicon wafer and left to dry for several hours. The 4-NTP molecules were self-assembled by immersion of the nanoflower-fabricated wafer in an ethanolic solution of 5 mM of 4-NTP for 6 h. The wafer was then washed with ethanol and water to remove the unattached molecules before being measured.

### Characterization

AuNFs were placed on a carbon-coated copper grid and imaged by transmission electron microscope (JEM-1011, JEOL Japan) operated at an acceleration voltage of 80 kV. A typical TEM image of the particles is shown in Fig. 1b. The extinction spectrum of the AuNFs was measured using a standard UV-Vis spectrometer (VIRIAN CARY 5000) Fig. 1b, inset).

Raman spectra were measured using a confocal Raman microscope (alpha 300; WITec, Ulm, Germany) equipped with a laser excitation of a wavelength at 785 nm. The laser wavelength was chosen such that it lies well separated from any direct vibrionic excitation of the molecules, but still within the plasmon band of the particles. This way photoreactions by direct excitation of the reactant can be exclude. The laser beam was focused on the sample through 10 × (Nikon, NA = 0.25) microscope objective. The spectra were acquired with a thermoelectrically cooled CCD detector (DU401A-BV, Andor, UK) placed behind the spectrometer (UHTS 300; WITec, Ulm, Germany). A Raman band of a silicon wafer at 520 cm^−1^ was used to calibrate the spectrometer. For the temperature dependent Raman measurements, the sample was placed in a closed chamber of a Linkam heating stage where the laser was focused through a transparent window into the sample.

The Raman thermometry was performed by simultaneously recording the Stokes and anti-Stokes Raman spectra using a confocal Raman microscope (JASCO NRS-4100) coupled with a laser excitation of a wavelength of 785 nm at different excitation powers. An intensity calibration delivered by the manufacturer was used to correct the wavelength-dependent sensitivity of the silicon CCD. Different excitation powers were achieved using neutral density filters with a transmission of 0.01%, 0.1% and 1%. That corresponds to laser powers of 20 µW, 150 µW and 1 mW, respectively.

## Results and Discussion

**Figure 2:**
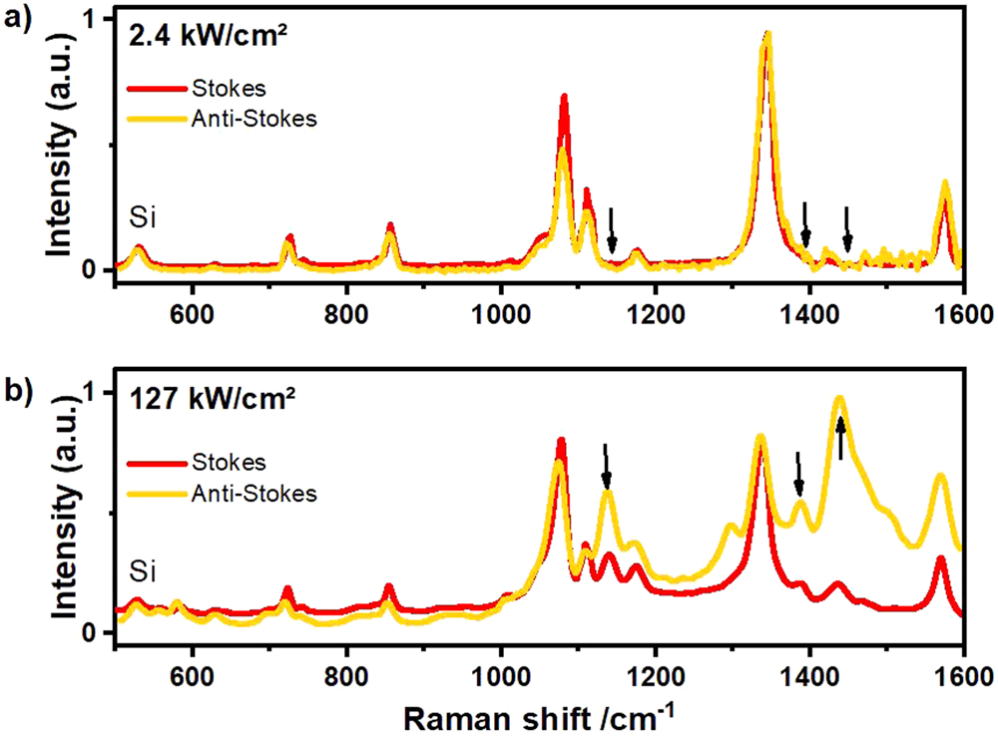
SERS spectra of 4-NTP recorded in both the Stokes and the anti-Stokes regions for an intensity of (**a**) 2.4 kW/cm^2^ and (**b**) 127 kW/cm^2^. The anti-Stokes region has been temperature adapted using the temperature of the 1332 cm^−1^ peak for comparison to the Stokes spectrum as discussed in the text.

Gold nanoflowers (AuNFs) with a diameter of about 60 nm ± 10 nm shown in (Fig. 1b) were prepared using a facile method, where the HEPES buffer was used as the structural-directing and reducing agent. 4-NTP molecules were self-assembled on the surface of the deposited nanoflowers. Plasmonic nanostructures such as the nanoflowers were reported to exhibit a high SERS enhancement owing to their tips, at which the electric field and therefore Raman scattering is enhanced^32–36^. The high signal enhancement enables monitoring of the plasmon-driven dimerization of 4-NTP on both the Stokes and the anti-Stokes spectral range in real time as the reaction proceeds. (Fig. 2a) shows Stokes and anti-Stokes SERS spectra of 4-NTP recorded with low intensity (2.4 kW/cm^2^), with no indication of the reaction product. The spectrum is dominated by the main Raman peaks of 4-NTP at 1082, 1332, and 1575 cm^−1^, assigned to the C−H bending, NO_2_ symmetric stretching, and C = C stretching modes of 4-NTP, respectively^37^. Increasing the laser intensity (127 kW/cm^2^) induces the dimerization reaction as evidenced by the Raman peaks of DMAB. (Fig. 2b) displays SERS spectra of 4-NTP measured with an integration time of 1 second, showing the reaction product in both the Stokes and the anti-Stokes regions, where peaks at 1134, 1387, and 1434 cm^−1^ assigned to the C−N symmetric stretching and N=N stretching vibrational modes of DMAB (marked with black arrows)^38,39^.

**Figure 3:**
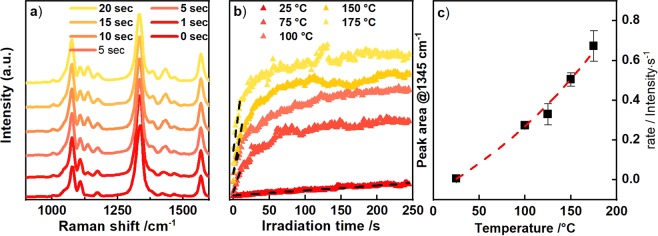
(**a**) SERS spectra of 4-NTP after different irradiation times with a laser intensity of 25.5 kW/cm^2^ (**a**) at a bath temperature of T = 293 K. (**b**) Kinetics of the product extracted from the peak area at 1345 cm^−1^ in a) for different temperatures. The NT temperature is around 75 K higher than the external heating temperature indicated in the legend. (**c**) Rates extracted from kinetics in (**b**) (fitting indicated in (**b**) by dashed lines).

From the intensity ratio ${I}_{AS}/{I}_{S}\,\,$ of the anti-Stokes and Stokes SERS spectra we determined the temperature $\,{T}_{vib}=\frac{-h\nu }{{k}_{B}\,\mathrm{ln}(\frac{{I}_{AS}}{C\cdot {I}_{S}})}$ for the vibrational band at the frequency $\nu$ during the reaction^40^. Here, $C={(\frac{{\nu }_{L}+\nu }{{\nu }_{L}-\nu })}^{4}$ describes the dependence of the Raman scattering on the laser frequency ${\nu }_{L}$. The temperature of the 4-NTP bands increases from room temperature for excitation with 2.4 kW/cm^2^ and 127 kW/cm^2^ by about $\Delta {T}_{vib,4NTP}=60\,K$ and 200 *K*, respectively. As we will discuss later in Fig. 3b, 200 *K* temperature increase during the reaction by stationary heating strongly enhances the reaction rate.

In order to visualize the accuracy of the temperature assignment, we plot the Stokes Raman signal on top of an anti-Stokes Raman signal, which has been intensity-scaled according to $\,{I}_{AS}=C\cdot {I}_{S}{e}^{\frac{h\nu }{{k}_{B}{T}_{vib}}}$ after subtracting a constant background. Subtracting inelastic light scattering according to the Fermi-Dirac statistics does not considerably change our conclusion^29,41^. More important is the striking systematic intensity increase of the DMAB bands in the anti-Stokes spectra which in fact would be consistent with a considerably higher vibrational temperature rise of ${\rm{\Delta }}{T}_{vib,DMAB}=350K.$ A possible explanation for this temperature difference between reactant and product is that the reaction preferentially proceeds at the hot spots.

To compare the vibrational temperatures of the molecule to the electron temperature of the nanoparticle, we made use of the observation by the Boerigter *et al*. that the anti-Stokes background intensity measures the rate anti-Stokes shifted photons scattered from the Fermi-Dirac distributed electrons in the nanoparticle^29^. Following their procedure, we fitted the anti-Stokes background by ${I}_{AS,bg}={I}_{0}{[{e}^{h\nu /{k}_{B}T}+1]}^{-1}$, were ${I}_{0}$ is the background at $\nu =0$. At $2.4\,\mathrm{kW}/{{\rm{cm}}}^{2}$ excitation the temperature rise of the particle was with ${\rm{\Delta }}{T}_{NP}\approx 60\,K$ approximately identical to the vibrational temperature of the molecules. For the higher excitation intensity of $127\,\mathrm{kW}/{{\rm{cm}}}^{2}\,$, on the other hand, the electron temperature of the particle rises with ${\rm{\Delta }}{T}_{NP}\approx 150\,K$, which is significantly less than the temperature of both 4NTP and DMAB, but in good agreement with recent measurements of the phonon temperature of the nanoparticles under laser irradiation by X-ray diffraction^26^. In both high and low intensity measurements, the particle temperature is roughly identical with the temperature of the silicon substrate, which could be determined from the prominent Si peak at ${\nu }_{Si}=550c{m}^{-1}$. Evidently, the molecular vibrational occupation is not in equilibrium with the particles, while the particles are in thermal equilibrium with the substrate phonons.

Since the particle temperature is approximately identical to the substrate temperature, we investigated the influence of the temperature on the product formation, by time-dependent SERS measurements recorded while heating the substrate with an external heater to $25,\,75,\,100,\,150$ and 175 °C, respectively. The sample was held in a closed chamber of the Linkam stage and the temperature was automatically controlled. The temperatures were chosen, such that the higher temperatures were similar to the particle temperatures obtained by pure laser heating. Despite the similar particle temperatures, the observed reactant temperatures could not be reproduced by external heating of the sample, since for ${\rm{\Delta }}{T}_{heater}\ge 175\,K$ the particles started to melt. By using an intensity of $25.5\,\text{kW}/{{\rm{cm}}}^{2}$, the lowest intensity for which we obtained a clearly visible DMAB signal, we kept laser heating to the minimum possible. From the values for laser heating discussed earlier, we estimate an additional laser heating at this intensity of roughly ${\rm{\Delta }}{T}_{Laser}=75\,K$.

We exemplify the evolution of the reaction product at room temperature by SERS spectra taken after different laser irradiation times (Fig. 3a). The increase of the peak area at 1134 cm^−1^, i.e. the formation of the number of reaction products, is shown for all bath temperatures in Fig. 3b. We estimated the temperature dependence of the kinetic rate, by fitting the initial product increase with a linear function (Fig. 3c). The zero-order rate extracted this way follows an exponential dependence of the temperature indicating that the reaction has an Arrhenius-type behavior. The activation energy however cannot be calculated without knowing the Raman cross-section of the reactant molecules. Qualitatively, the rate-temperature plot shows that heating the setup by $\,{\rm{\Delta }}{T}_{heater}=150\,{\rm{K}}$ enhances the reaction rate by two orders of magnitude.

Simultaneous to DMAB peak increase, the NO_2_ Raman peak of the 4-NTP at 1332 cm^−1^ gradually decreases, confirming the loss of reactants required for the dimerization reaction (Fig. 3a). However, when the intensity of the DMAB peak is saturated about 70% of the 4-NTP intensity remains. Thus the reaction can only proceed for a small fraction of the molecules on the AuNF surface, and probably the SERS enhancement even biases our observation towards those molecules which receive the highest light intensity. This observation further strengthens the argument that the reaction indeed takes place at hot spots only.

**Figure 4:**
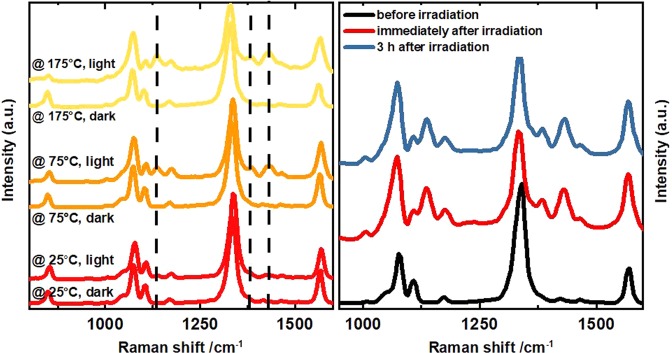
(**a**) Temperature dependent SERS of 4-NTP measured with 2.44 kW/cm² intensity Raman laser after keeping the sample spot under dark (no light) and bright (25.4 kW/cm² for 5 min) conditions. SERS spectra measured with an intensity of 2.4 kW/cm². (**b**) SERS spectra of 4-NTP confirming the stability of the DMAB product.

Since temperature turns out to be an important parameter for the reaction, we attempted to drive the reaction solely by supplying heat. The sample was heated by the external heater stage keeping the sample in the dark. (Fig. 4a) displays temperature-dependent SERS spectra captured with very low excitation intensity and integration time ($2.4\,kW/c{m}^{2}$ and 0.5 s), after keeping the sample for 5 min at constant elevated temperature in the dark. These measurement conditions guarantee that the dimerization of 4-NTP can only be driven by heat, while the influence of the laser on the reaction process is kept to a minimum. None of these dark measurements in Fig. 4a show any signature of DMAB formation. Even considering the laser heating (${\rm{\Delta }}{T}_{laser}=75\,K$), a clear reaction was observed in Fig. 3b at the same nanoparticle temperature.

A further control sequence of SERS spectra taken under the same conditions, except that during the 5 min the sample was continuously irradiated by $25.5\,\text{kW}/{{\rm{cm}}}^{2}$, show pronounced peaks related to the reaction product (DMAB) at high temperatures (Fig. 4a). In a second control experiment we irradiated the solution of the 4-NTP molecules without the gold nanoflowers with high laser intensity for a long irradiation time. No reaction product bands were observed (NOT SHOWN).

Finally, we confirm that DMAB is a stable product in the dark and the reaction is not reversible when the light is switched off for several hours. The SERS spectrum is unmodified when switching the laser off for 3 hours as shown in (Fig. 4b).

Combining Figs 2 and 4 we see that a room temperature experiment, where light-driven heating leads to a nanoparticle heating of about $150\,K$, shows a large reaction yield within 1 second, whereas keeping the system at $175\,K\,\,$in the dark for 5 minutes does not produce the faintest signature of the product. These data confirm that the dimerization reaction is initiated by photons, probably by providing energetic electrons which are not thermalized to the Au lattice temperature^42,43^. The heating of the Au lattice and the molecular vibrations by a heating stage or by absorption of the photons by the particle only enhances the reaction rate.

To summarize, our experiments show that the dimerization reaction of 4-NTP on AuNFs cannot be triggered by normal heating. The AuNFs melt at temperatures above $473\,K$ and the Raman signal breaks down (NOT shown). If the AuNFs are heated by laser irradiation, the reaction proceeds although the vibrational temperature of the Au lattice and of the molecules stays below $473\,K$. This suggests that the electron system in the AuNFs is not in a thermal equilibrium with the lattice, thus providing energetic electrons which can initiate the reaction. It is experimentally very difficult to precisely investigate this reaction in a systematic sequence of experiments, since it is irreversible and each new spot on the plasmonic template may have different hot spots, which will lead to a broad distribution of field enhancement. Therefore, we could not check the functional dependence of the reaction rate on the light intensity.

**Figure 5:**
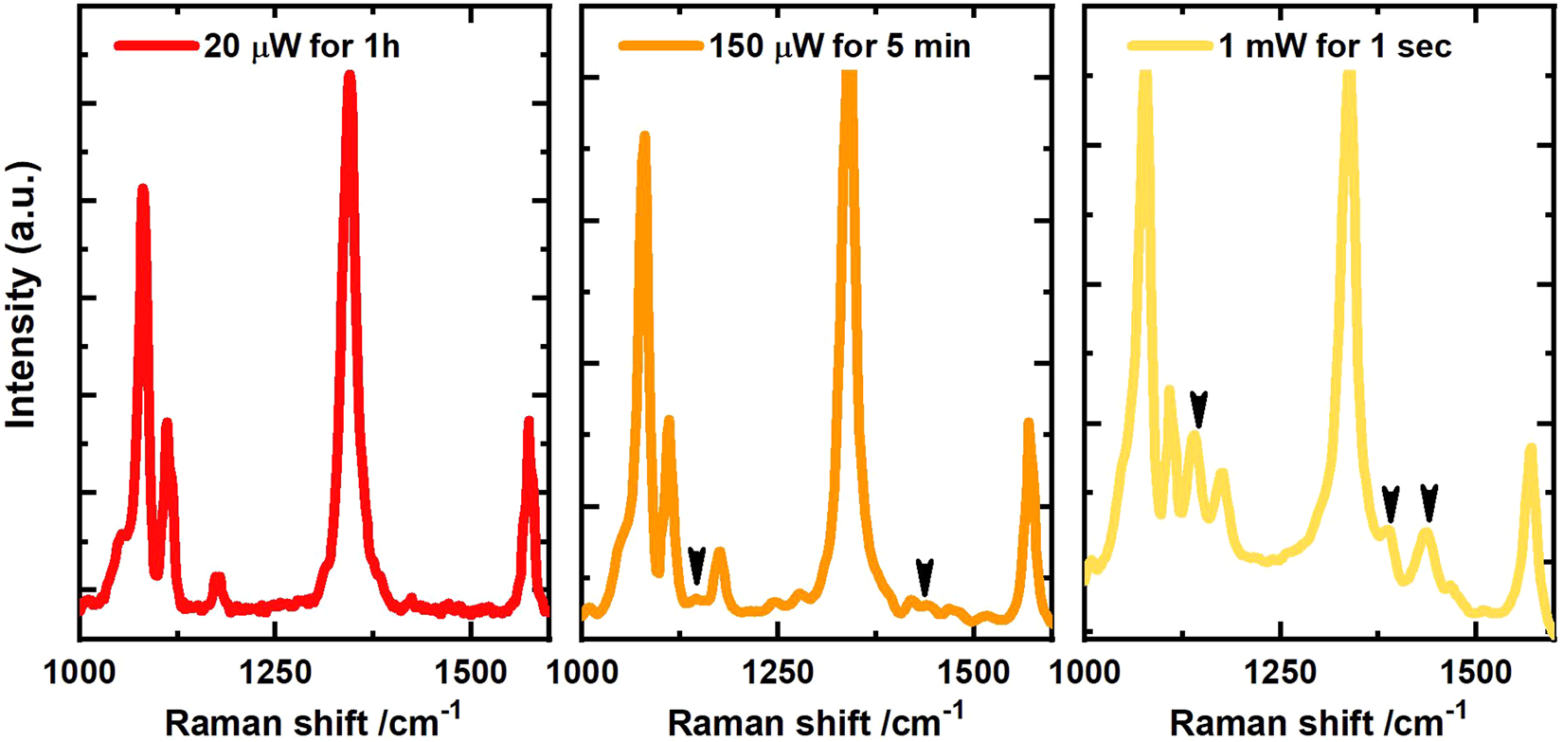
Comparing the product yield for the same number of photons and reactants. (**A**,**B**) show spectra recorded with the same integral photon number, however, at 10 times different power and integration time. (**C**) shows a spectrum after irradiation with 30 times less photons, however, at 10 times increased laser power.

To give at least some quantification of the strongly nonlinear character of the reaction, we show in Fig. 5A series of experiments at room temperature, where spots with similar field enhancement have seen a similar total number of photons, however at drastically different intensities. Figure 5A confirms the absence of DMAB for an intensity of $2.4\,kW/c{m}^{2}$ even after irradiation for 60 min. Increasing the laser intensity by a factor of 8 ($19\,kW/c{m}^{2}$) and reducing the irradiation time by the same factor yields tiny DMAB peaks already. Increasing the intensity by another factor of 7 $(127.4\,kW/c{m}^{2})$ yields a dramatic increase of the signal after 1 second, although the same integral photon number would be reached after 30 seconds.

In order to rationalize that there might be a non-thermalized electron distribution, which either does not have the same temperature as the lattice and the molecular vibrations, or may even not be described by a Fermi-Dirac distribution, we recall the electron-phonon coupling time of 1 ps typically observed in Au nanoparticles by femtosecond laser-spectroscopy^25^ or ultrafast x-ray diffraction^44^. Next we calculate that under the relevant intensity of 240 kW/cm^2^, each nanoparticle with a diameter of 70 nm is hit by a photon every 200 fs. Although not all photons impinging on the nanoparticle are absorbed, we believe that at least around hot spots of the plasmonic structure, the photons are absorbed faster than their energy is dissipated by *e-ph* interaction.

## Conclusion

In summary, we have observed the temperature dependence of the dimerization reaction of 4-NTP to DMAB. Simultaneous measurement of the Stokes and the anti-Stokes regions of the SERS spectrum enabled the *in-situ* temperature measurements under the reaction conditions. The average temperature of the product is larger than that of the reactant because the reaction proceeds preferentially at hot spots. Both measured vibrational temperatures exceed the temperature of the nanoparticles crystal lattice, in good agreement with previous findings^29^. We think that the temperature of the electrons and lattice at the Au tips is reduced on the femtosecond timescale by heat transport within the metal, whereas the cooling of the molecules proceeds slower by vibrational interactions. This corroborates the hypothesis that hot electrons emitted from the tips trigger the dimerization reaction, since the temperature within Au is smaller than the molecular temperature and vibrational heat alone could be ruled out as the driving mechanism. Nonetheless, the reaction rate can be increased by an order of magnitude by stationary heating of 150 K, which implies that the effect of vibrational temperature cannot be neglected when analyzing this plasmon driven reaction. Clearly it is not the total number of absorbed photons which is relevant but rather the collective action of several photons is required for triggering the reaction.

## Data Availability

The datasets generated and analyzed during the current study are available from the corresponding author on reasonable request.
